# Rapidly, sensitive quantitative assessment of thiopental via forced stability indicating validated RP-HPLC method and its in-use stability activities

**DOI:** 10.1038/s41598-023-37329-0

**Published:** 2023-06-25

**Authors:** Mostafa F. Al-Hakkani, Nourhan Ahmed, Mohammad H. A. Hassan

**Affiliations:** 1 Department of Chemistry, Faculty of Science, Al-Azhar University, Assiut Branch, Assiut, 71524 Egypt; 2 Department of Research, Development, and Stability, UP Pharma, Industrial Zone, Arab El Awamer, Abnoub, 76, Assiut, Egypt; 3 Department of Medical Laboratory Technology, Higher Technological Institute for Applied Health Sciences in Minya, Minya, Egypt

**Keywords:** Analytical chemistry, Green chemistry, Inorganic chemistry

## Abstract

Thiopental sodium (Tho) is an intravenous anesthetic. The current study aimed to find a rapid RP-HPLC method of Tho analysis with high linearity, repeatability, sensitivity, selectivity, and inexpensive. In our developed method, there is no need to use special chemical reagents, a high percentage of organic solvent, a high flow rate, or a further guard column. The chromatographic system consists of an ODS column (150 mm × 4.6 mm × 5 μm). The mobile phase was prepared by mixing KH_2_PO_4_ solution: methanol (40:60) with a flow rate of 1.2 mL/min at a detection wavelength of 230 nm, at room temperature using an injection volume of 10 μL. The method manifested a satisfied linearity regression R^2^ (0.9997) with a good repeatability precision range (0.16–0.47%) with LOD and LOQ; 14.4 μg/mL and 43.6 μg/mL respectively. Additionally, the method proved its efficiency via system suitability achievement in robustness and ruggedness, according to the validation guidelines. The shorter analysis time makes the method very valuable in quality control to quantify the commercial Tho in pharmaceutical preparations. This improved HPLC method has been successfully applied for Tho analysis for Thiopental UP Pharma 500 mg vials and Thiopental Eipico 1.0 g vials in our routine finished and stability studies testing laboratories. Additionally, the detection limit of Tho has been tested in our quality control lab to detect the smallest amount of traces that may be present after the cleaning process of the production machines for cephalosporins preparations. The method has shown positive results for Tho in low-level raw materials and pharmaceutical formulations.

## Introduction

**Figure 1:**
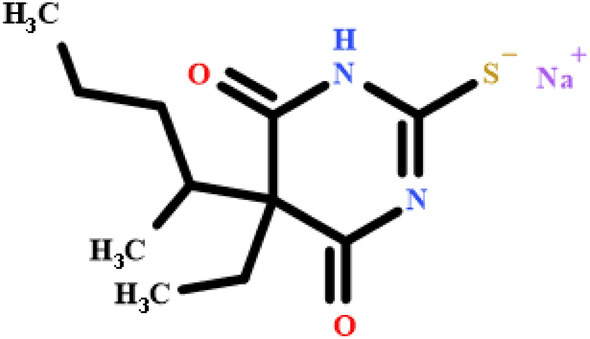
Structure of Thiopental sodium “C_11_H_17_N_2_O_2_SNa in molar mass “264.32 g/mole”.

Thiopental sodium (Tho) has the IUPAC name 4,6(1H,5H)-Pyrimidinedione, 5-ethyldihydro-5-(1methylbutyl)-2-thioxo-, monosodium salt, (±)-; Sodium (±)-5-ethyl-5-(1-methylbutyl)-2-thiobarbiturate^1^. Tho has the chemical molecular formula C_11_H_17_N_2_O_2_SNa in molar mass “264.32 g/mole” as manifested in (Fig. 1).

Tho could be taken by injection as intravenous anesthesia. It could be used for the induction of general anesthesia, as well as relaxation during balanced anesthesia with other anesthetic drugs such as analgesics and muscle relaxants^2^. As a reinforcement for the management of refractory convulsive disorders that are genetically diverse, such as those produced by local anesthetics. It is also used to minimize intracranial pressure in individuals who have high intracranial pressure if managed respiration is administered. Tho also has great importance in forensic medicine and criminology as it is used in investigations related to drug crimes^3^.

Tho is sold under more than one brand name, such as Farcopental, Thiopental, and Anapental, with different strengths of 1.0 g and 0.5 g vials. Tho also be found in a sterile mixture of thiopental sodium with or without anhydrous sodium carbonate as a buffer. Its finished product vial container must contain 93.0–107.0% of the labeled amount of thiopental sodium of the mixture according to the United States-Pharmacopoeia (USP 44–NF 39 2021). But the British-Pharmacopoeia (BP–2022–VOL3) issued another limit of the Tho assay as pure thiopental at 77.0–92.0% of the stated amount of the mixture of thiopental sodium and anhydrous sodium carbonate in the finished product vial container^1^.

The wide spectrum of this drug makes it important in the field of pharmaceutical trade, which necessitates the need to find effective, simple, easy, and rapid methods for assay determination. In addition, a sensitive method should be conducted at low concentrations of this drug preparation when this method is used to estimate Tho after washing, cleaning machines, and production lines. The sensitive method should be conducted to ensure the effectiveness of the cleaning method to remove the drug residual effects of this drug that may be entered into the next product in the production process, causing a completely unacceptable cross-contamination process^4–14^. This type of contamination is according to the quality standards mentioned in the rules of good manufacturing practice (GMP)^5,6,14^.

Label-free quantitation using an HPLC conjugated with their detectors is one of the simplest and most economical and cost-effective methods to differentiate the expression of proteins and their byproducts as metabolites. In label-free technology, each sample is measured separately. The necessity for high recovery and accuracy performance label-free quantitation methods is still high in the bioactive constituent, especially in the drug research field^15^.

There are many different methods with more than one technique in the analysis tools being conducted for the assay determination of Tho, some of which include the electrochemical method of the glassy carbon electrode by cyclic voltammetry^16^, cathodic stripping voltammetry^17^, HPTLC^2^, GC^18–20^, GC–EI–MS method^21^, LC–MS/MS^22^, HPLC^3,23–28^, UV spectrophotometry as USP 44–NF 39 2021, gravimetric analysis as constant weight using chloroform for the extraction as BP–2022–VOL3 issued^1^.

Most of the disadvantages of the previous analysis techniques and methods were detailed as more expensive^2,18,19,21,25^, time-consuming^1,20^, hazardous as chloroform^1,6,26^, sodium fluoride^22^, iodine-azide reaction^27^, needing special conditions or reagents as tetrabutyl ammonium bromide^26^ or using pre-column^20,28^, EDTA, CN column, sodium hydroxide as a solvent, and post-column^27^, sometimes derivatization was required. Also, they lack sufficient selectivity and specificity, especially with UV methods, and adjusted pH buffer solutions^22,25,27^. These factors are used to get the optimum peak shape with ideal tailing^29,30^. Supplementary Table 1 shows a brief comparison of different assay methods including the important parameters.

The field of scientific research has recently tended to purify industrial wastewater, pharmaceutical factories, and hospitals. So, finding easy, fast, sensitive, accurate, and economical methods has become an urgent necessity^4–10,14,27,31–33^.

The current paper aims to introduce an inexpensive, efficient, simple, and rapid method for the assay determination of Tho. It is because of the scarcity of quick and simple analytical methods to determine the thiopental assay in recent years that most of the system suitability and method validation parameters have been implemented. Furthermore, inexpensive chemicals with integrational analysis method parameters were used to obtain the detection and identification of Tho with accurate selectivity and high specificity via reasonable resolution.

## Methodology and experimental analysis

Thiopental sodium and sodium carbonate working standards were supplied as a gift sample from UP Pharma (Assuit, Egypt). Methanol HPLC-grade, potassium dihydrogen phosphate, hydrochloric acid (37%), sodium hydroxide, and hydrogen peroxide (30%) (Scharlau, Spain). Water for injection (WFI) was used in the analysis and passed through a 0.45 μm nylon membrane filter before use. A phosphate solution was prepared by weighing about 5.82 g of potassium dihydrogen phosphate in 1000 mL of WFI.

### Chromatographic system configuration

Compared with the previously conducted HPLC methods and the current analysis method, we did not use a dedicated pH solution adjustment or special chemical reagent to realize the optimum separation for the ideal system suitability achievement.

Tho assay determination was conducted using the HPLC model HP 1100 series with a variable wavelength. The current method was conducted with the RP C18 ODS column (150 mm × 4.6 mm × 5 μm) (Thermo Scientific). The mobile phase was prepared as KH_2_PO_4_ solution “(5.82 g in 1000 mL of WFI)”: methanol in a ratio of 40:60 v/v, at a flow rate of 1.2 mL/min with a detection wavelength of 230 nm at room temperature and an injection volume of 10 μL.

### Parameters of method validation

The HPLC validation method was performed according to the International Conference on Harmonization (ICH) guidelines concerning parameters including system suitability, range of linearity, the limit of detection (LOD), the limit of quantification (LOQ), repeatability (precision), recovery and accuracy, robustness, ruggedness, the stability of the solution, specificity, and selectivity^11–13,34–36^.

### System suitability check

System suitability was performed by injecting six replicate injections of the same working standard solution, which was prepared by dissolving a quantity of thiopental sodium with a sodium carbonate mixture of about 1.080 g, equivalent to 1.0 g of Tho [stock solution], in 200 mL of WFI, then diluting 10 mL into a 100 mL volumetric flask using WFI to get a solution with a concentration about 500 µg/mL.

### Range & linearity

The analytical approach is deemed to be linear if there is a substantial portion between the response and the claimed working concentration, starting at the lowest point in the tested range and increasing to the highest point with R^2^ ≥ 0.999^5,6,11–14,34,35,37^.

Regression linearity equation:

1 $$ {\text{Y }} = {\text{ a X }} \pm {\text{ b}} $$

where Y represents the response of the average peak area, X represents the claimed working concentration in (%), a represents the slope, and b is the intercept of the calibration curve where the target concentration (100%) represents 500 µg/mL.

The linearity parameter was submitted using different concentrations in the range (20–150%) of the Tho working standard. The claimed working concentrations were prepared using WFI as a solvent from the stock solution at a concentration of 5000 µg/mL. Every solution was injected into duplicates.

### Limit of detection (LOD)

It was defined as the lowest specified analyte concentration in the matrix that could be identified using the detection of the instrument. Furthermore, it should not be included in the accuracy, precision, and linearity ranges^11–13,34,35^.

### Limit of quantitation (LOQ)

It was defined as the lowest specified analyte concentration in the matrix that could be identified using the detection of the instrument. Furthermore, it must be included in the accuracy, precision, and linearity ranges^11–13,34,35^.

LOD and LOQ could be calculated according to the slope and standard error data from the linearity of the calibration as follows:

2 $$ {\text{LOD}}\; = \;3.3\;\upsigma /{\text{S}} $$

3 $$ {\text{LOQ}}\; = \;{10}\;\upsigma {\text{/S}} $$

where σ: is the standard error of X & Y arrays and S: represents the slope of the linearity calibration curve.

### Accuracy and recovery

Both recovery and accuracy are used alternately. The measurement's accuracy is defined as the proximity of the actual concentration (measured value) to the theoretical concentration (true value)^11–13,34,35^.

Accuracy was implemented by weighing three individual Tho working standards to give theoretical concentrations at (350 µg/mL), (500 µg/mL), and (600 µg/mL). Accuracy% could be estimated using the linearity equation:

4 $$ {\text{Accuracy\% }}\; = \;{\text{ Actual}}\;{\text{ Conc.}}{\text{\% /Theoretical}}\;{\text{ Conc.}}{\text{\% }} \times \, 100 $$

5 $$ \begin{aligned} {\text{Actual }}\;{\text{Assay \% }} & \; = \;{\text{ Peak}}\;{\text{ area}}\;{\text{ of}}\;{\text{ the}}\;{\text{ test/mean }}\;{\text{peak}}\;{\text{ area}}\;{\text{ of }}\;{\text{the }}\;{\text{working }}\;{\text{standard }} \\ & \;\;\; \times {\text{ working }}\;{\text{standard }}\;{\text{weight/test }}\;{\text{weight }} \times \, 85.75\% \\ \end{aligned} $$

Where 85.75% is the working standard assay as is%

### Repeatability and precision

Repeatability was conducted using six different preparations individually of the target concentration of the intended method 100% (500 µg/mL) of Tho using the same equipment on the same day via the same analyst or compared with another analyst as inter–precision^11–13,34,35,38^.

### Robustness

Robustness was submitted using designed small changes including slight changes, in the temperature, composition of the mobile phase, etc.

The designed small changes were conducted in a different organic solvent ratio (Methanol) at (600 ± 60 mL) and a flow rate of 1.2 mL/min ± 0.1 mL/min).

### Ruggedness

Ruggedness was submitted using designed and major observable changes, including analyst-to-analyst, column-to-column, and day-to-day while maintaining all of the analysis method parameters and conditions as they are without changes.

### Stability of sample solution (at the HPLC rack)

This test was performed to judge the stability of the solution after preparation over time. The test was conducted at the target concentration of 100% (500 µg/mL) over 48 h. The test is valid for use at room temperature over 48 h if the RSD% of the peak area is ≤ 2.0% from the start to the end of the conducted experiments.

### Specificity and selectivity

Forced degradation studies were performed to indicate the stability-indicating properties. Accelerated degradation was implemented using acid hydrolysis at 0.1 M HCl for 30 min, base hydrolysis at 0.1 M NaOH for 30 min, stress oxidation degradation at 3.0% w/v of H_2_O_2_ for 30 min, and light degradation over 3 h.

### Test of the validated method

#### Tho analysis of the different commercial dosage forms in the Egyptian local market

Thiopental UP Pharma 500 mg vials and Thiopental Eipico 1.0 g vials were tested using the validated method of Tho.

#### Thiopental UP pharma 500 mg vials batch number (220741) after the constitution stability studies

The after-constitution stability study was conducted using the WFI, saline 0.9% wt/v, and glucose 5% wt/v at zero time, 6 h at room temperature 30 ± 2 °C, and in the refrigerator at temperature 5 ± 3 °C for 24 h.

The constituted vial was performed using 10 mL of solvent, and the constituted vial was reserved for the desired time under the described storage conditions. Then, all of the content of the vial was transferred into a 1000 mL volumetric flask, completed to the mark using WFI to get a solution concentration (0.5 mg/mL), and introduced to the HPLC assay.

## Results and discussions

### System suitability check

**Figure 2:**
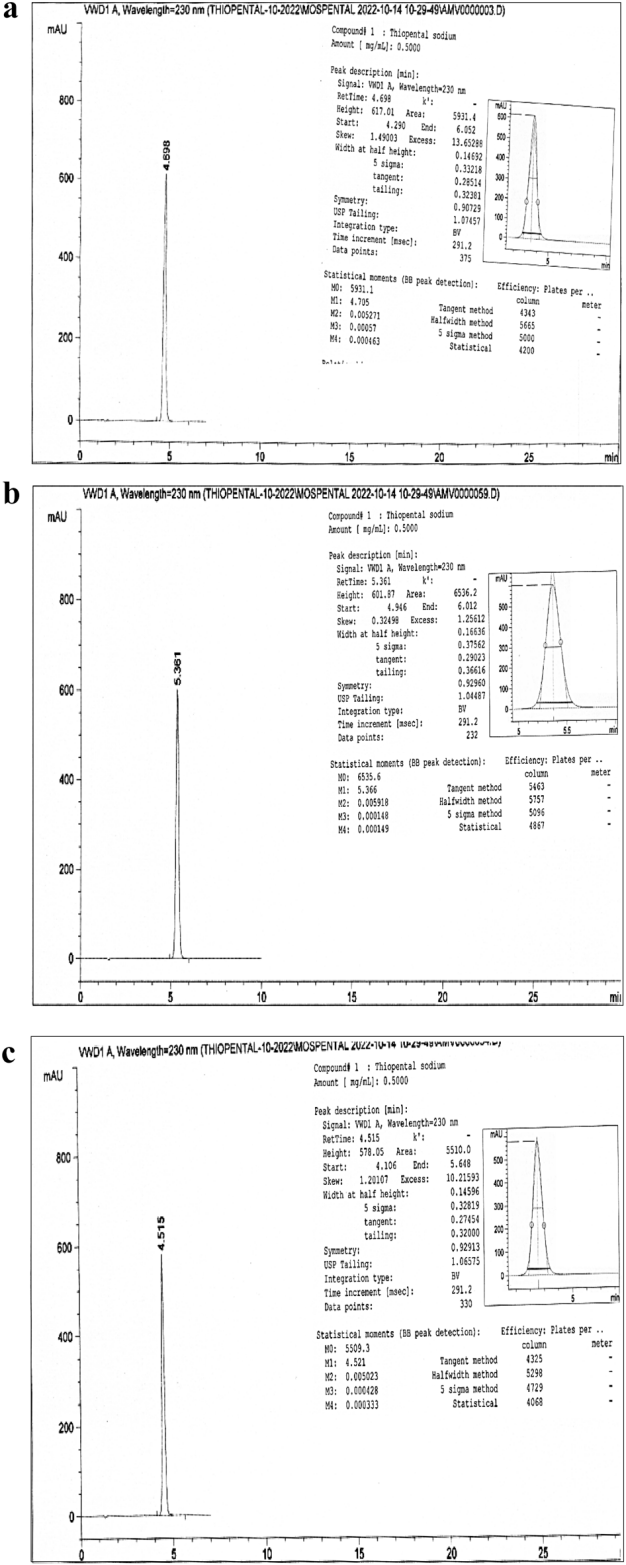

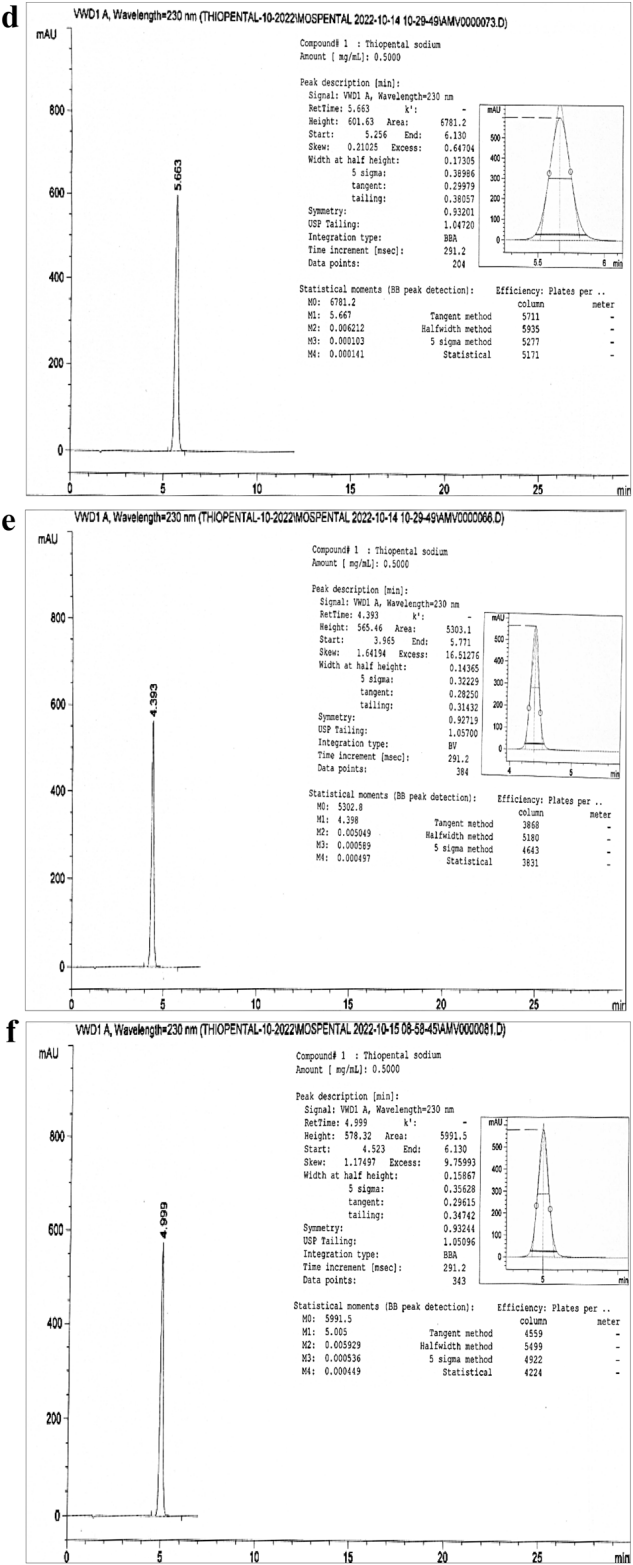

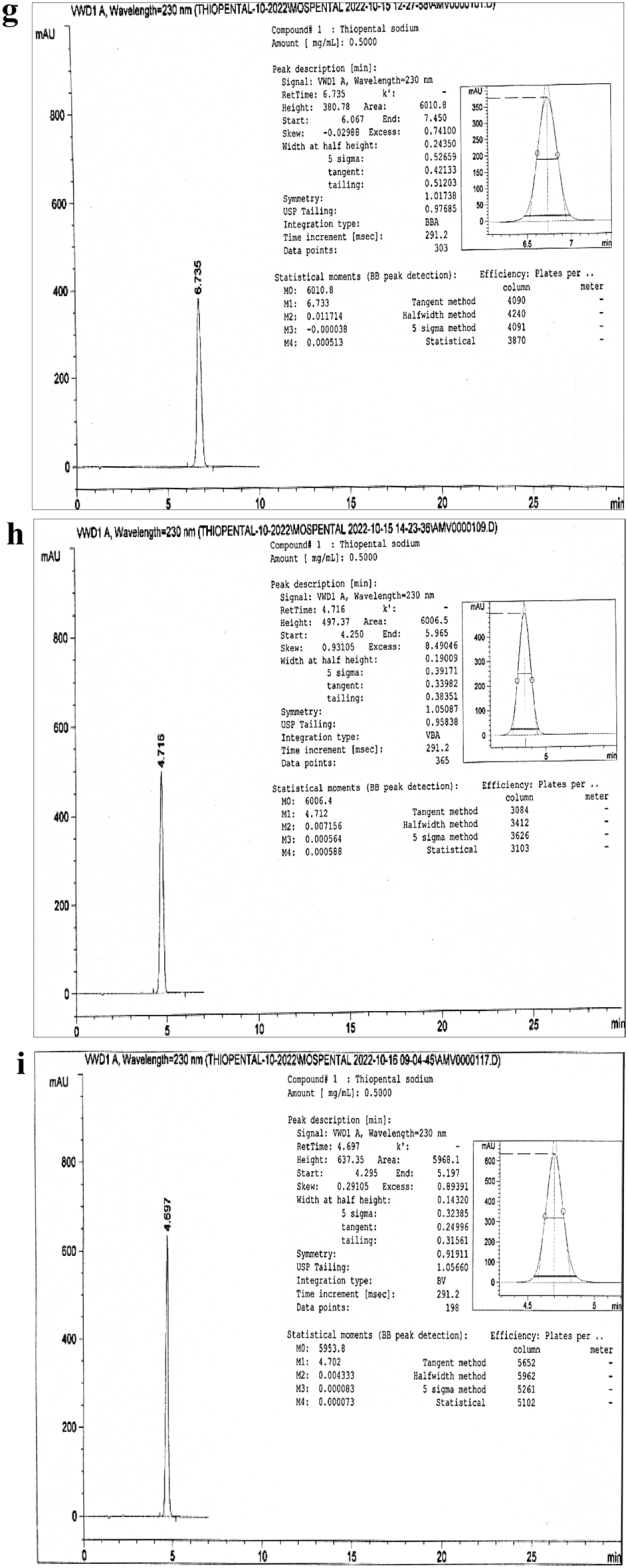
(**a**) Tho chromatogram at a flow rate 1.2 mL/min, Column—1, Aqueous phase 40% and Methanol 60%. (**b**) Tho chromatogram at a flow rate 1.1 mL/min, Column—1, Aqueous phase 40% and Methanol 60%. (**c**) Tho chromatogram at a flow rate 1.3 mL/min, Column—1, Aqueous phase 40% and Methanol 60%. (**d**) Tho chromatogram at a flow rate 1.2 mL/min, Column—1, Aqueous phase 46% and Methanol 54%. (**e**) Tho chromatogram at a flow rate 1.2 mL/min, Column—1, Aqueous phase 34% and Methanol 66%. (**f**) Tho chromatogram at a flow rate 1.2 mL/min, Column—1, Aqueous phase 40% and Methanol 60%, Day—2. (**g**) Tho chromatogram at a flow rate 1.2 mL/min, Column—2, Aqueous phase 40% and Methanol 60%, Day—2. (**h**) Tho chromatogram at a flow rate 1.2 mL/min, Column—3, Aqueous phase 40% and Methanol 60%, Day—2. (**i**) Tho chromatogram at a flow rate 1.2 mL/min, Column—1, Aqueous phase 40% and Methanol 60%, Day—3.

The thiopental peak appeared about 4.6 min at the optimum parameters of the analysis method (Fig. 2a) and in the range of 4.3–6.7 min over all the parameter changes (Fig. 2b–i). Table 1 showed high performance for the intended analysis method, where the RSD % < 2.0%, USP tailing < 2.0, and theoretical plates ≥ 2000^5,6,11–14,34,35^. So, according to the output data of the system suitability parameters, the method manifested superior validity through a wide range of retention times.

**Table 1:** System suitability.

Replicate	Flow (1.2 mL) Methanol 600 mL First analyst Day—1 Column—1	Flow (1.3 mL)	Flow (1.1 mL)	Methanol (540 mL)	Methanol (660 mL)	Second analyst	Day —2	Day —3	Column —2	Column —3
Weight (mg)/100 mL	54.2	54.2	54.2	54.2	54.2	54.3	54.5	54.4	54.5	54.5
1	5931.4	5510.0	6536.2	6781.2	5303.1	5926.1	5991.5	5968.1	6010.8	6006.5
2	5944.4	5502.7	6515.0	6791.1	5299.4	5922.9	5986.2	5950.8	6008.6	6000.1
3	5943.1	5498.4	6525.3	6786.0	5293.0	5931.8	5994.6	5958.0	6013.7	5991.4
4	5925.0	5518.2	6544.8	6799.4	5287.8	5971.0	5989.5	5954.7	6030.6	5993.6
5	5940.2	5512.4	6511.7	6785.7	5318.9	6038.6	5988.9	5956.4	6026.0	5996.1
6	5915.2	5508.7	6582.4	6796.2	5290.2	5956.7	5987.7	5960.9	6014.8	6009.1
Mean P.A	5933.2	5508.4	6535.9	6789.9	5298.7	5957.9	5989.7	5958.2	6017.4	5999.5
STDV	11.5	7.0	26.0	6.9	11.4	43.8	3.0	5.9	8.8	7.1
RSD %	0.19	0.13	0.40	0.10	0.22	0.74	0.05	0.10	0.15	0.12
Tailing	1.07	1.07	1.04	1.05	1.06	1.07	1.05	1.06	0.98	0.96
Plates	5665	5298	5757	5935	5180	5665	5499	5962	4240	3412

Russo et al.^28^ reported their method for Tho determination earlier, where they used a high flow rate of 2.0 mL/min with 2 columns showing results without any data about the system suitability and a very short introduction to method validation parameters showing a long retention time of 8 min compared to our method. Despite their use of a low concentration in the calibration curve of 1–100 µg/mL. Also, they used two different injection volumes of 20 μL, and 50 μL to achieve high linearity.

Also, many authors introduced their papers to separate and determine the Tho assay in the past two decades, from 1979 to 1998, but the conducted methods were deficient and lacked the implementation of several method validation factors, especially system suitability. With all of these scientific contributions, we can’t deny their efforts. We show a rapid survey so the reader doesn’t get bored. These approaches manifested long at the retention time, absence of tailing factor, column efficiency, theoretical plates, resolution, specificity, recovery, theoretical plates, resolution, specificity, recovery, precision, or use of hazards, chemical reagents, and special conditions. Also, high flow rates, two columns, high or more than one organic modifier, or mobile phase pH adjustment may be used in the separation process, which is not present in our current method^39–57^.

The HPLC method by Beril et al.^3^ was introduced using acetonitrile (A), methanol (B), 50 mM pH = 2.7 phosphate buffer (C) as mobile phase in the ratio (40:5:55) of A: B: C (4.00 mL/min). The retention time was introduced as 10.57 (± 0.010) min, LOD and LOQ 0.56, 1.69 ppm with RSD repeatability of 4.98%. The method used a high flow rate and adjusted the buffer solution to more acidic conditions with two different organic modifiers, acetonitrile and methanol. Also, the method did not show any details about the system suitability parameters except for linearity, LOD, LOQ, and repeatability, with very bad repeatability results.

Coppa and his co-workers reported their HPLC method for Tho determination with a very shortfall in analytical method validation parameters, especially in selectivity, specificity, and system suitability. Additionally, they used a tertiary system of mobile phase consisting of two organic modifiers, methanol and acetonitrile, with a flow rate of 1.5 mL/min to get a retention time of about 9.5 min which is time-consuming compared with our method^23^.

Recently, Elmansi et al.^25^ reported a new method for Atracurium and Thiopental by HPLC/ UV method using the factorial model in one chromatogram where Tho LOD was 0.02 μg/mL. However, from our point of view, this method requires special conditions, such as using a high ratio of methanol to 80% of the mobile phase and needing to adjust the phosphate buffer to pH 6.5. Also, the method uses a high content of methanol as a solvent and further dilution to prepare the calibration solution. In addition, this method lacked the application of many parameters of the method validation, such as system suitability parameters such as tailing or symmetry factor, theoretical plates, RSD%, accuracy and recovery ruggedness, and specificity. Colatutto et al.^20^ also didn’t introduce satisfactory parameters for the method validation and didn’t mention a full analysis of the method parameters of the HPLC system in their paper.

### Range and linearity

**Figure 3:**
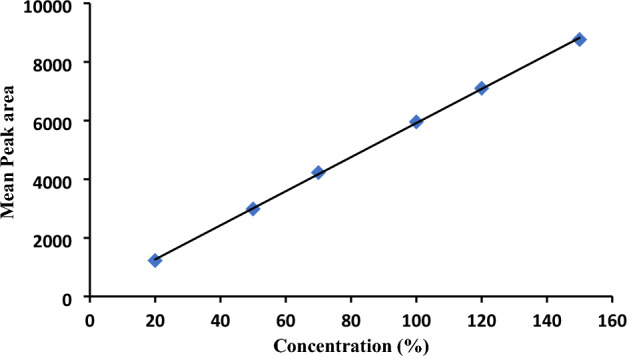
Linearity calibration curve of Tho.

The main aim of our study is the Tho assay determination in the lab in the pharmaceutical dosage form. The results manifested high linearity with R^2^ = 0.9997 at the working concentrations in the range (20–150%) as we can see in Fig. 3 and Table 2.

**Table 2:** Range and linearity.

Working conc (%)	Average P. As	Statistical data	
20%	1226.6	R^2^	0.9997
50%	2984.6	Slope	58.136
70%	4224.6	Intercept	100.49
100%	5952.8	Standard error	50.69
120%	7100.7		
150%	8762.8		

### LOD and LOQ

LOD and LOQ limits could be determined simply using the linearity calibration data of Tho. LOD was found to be 14.4 µg/mL whereas LOQ was 43.6 µg/mL.

### Accuracy and recovery

Table 3 showed that the accuracy results of the tested range (70–120% of the target concentration of 100% (500 µg/mL) were found to be within the acceptance criteria (98–102%)^5,6,11–14,34,35^. Compared with Russo et al.^28^, our method manifested high accuracy and recovery percentages of the intended method, whereas their method showed 87.9 ± 6.2% recovery with a poor percentage of 93.2% at 50 µg/mL.

**Table 3:** Accuracy and recovery.

Theoretical conc. (%)	Actual peak area	Mean peak area	Actual conc. (%)	Accuracy (%)
70	4222.4	4226.7	71.0	101.4
	4236.6			
	4221.2			
100	5960	5958.5	100.8	100.8
	5962.5			
	5953			
120	7114.6	7103.7	120.5	100.4
	7101.2			
	7095.2			

### Repeatability and precision

The RSD% of peak areas was used for judgment on the repeatability of the analyte using six different preparations at the same target at 500 µg/mL concentration as in Tables 4 and 5. It was found to be 0.39% and 0.34% within the intra–precision and 0.92% of inter–precision as it demanded in the repeatability requirements as RSD% < 2.0%^5,6,11–14,34,35^.

**Table 4:** Repeatability and precision “Inter precision (analyst to analyst)”.

Replicate #	Standard P.A					
1	5931.4					
2	5944.4					
3	5943.1					
4	5925.0					
5	5940.2					
6	5915.2					
Mean P.A	5933.2					
Weight (mg)	54.2					
Assay (%)	85.75					

Item	Analyst 1 P.A	Weight(mg)	Assay (%)	Analyst 2 P.A	Weight (mg)	Assay (%)
Determination-1	5931.1	54.2	85.7	5926.1	54.1	85.8
Determination-2	5991.3	54.3	86.4	5922.9	54.1	85.8
Determination-3	5986.4	54.3	86.4	5931.8	54.0	86.0
Determination-4	5994.4	54.2	86.6	5971.0	54.3	86.1
Determination-5	5937.4	54.2	85.8	6038.6	54.8	86.3
Determination-6	5921.9	54.1	85.7	5956.7	54.6	85.5
Assay mean (%)	86.1			85.9		
Assay STDEV	0.40			0.31		
Assay RSD (%)	0.47			0.36		
Pooled assay (%)	86.0					
Pooled STDEV	0.14					
Pooled RSD (%)	0.16

**Table 5:** Repeatability and precision “Intera precision (one analyst at 3 days)”.

Replicate #	Day 1	Day 2	Day 3						
	Standard P.A								
1	5931.4	5991.5	5968.1						
2	5944.4	5986.2	5950.8						
3	5943.1	5994.6	5958						
4	5925.0	5989.5	5954.7						
5	5940.2	5988.9	5956.4						
6	5915.2	5987.7	5960.9						
Mean P.A	5933.2	5989.7	5958.2						
Weight (mg)	54.2	54.5	54.4						
Assay (%)	85.75								

Item	Day 1 P.A	Weight (mg)	Assay (%)	Day 2 P.A	Weight (mg)	Assay (%)	Day 3 P.A	Weight (mg)	Assay (%)
Determination-1	5931.1	54.2	85.7	5926.1	54.1	85.5	5857.0	53.3	86.0
Determination-2	5991.3	54.3	86.4	5922.9	54.1	85.4	6040.8	54.4	86.9
Determination-3	5986.4	54.3	86.4	5931.8	54.0	85.7	5922.3	53.4	86.8
Determination-4	5994.4	54.2	86.6	5971.0	54.3	85.8	5853.6	53.2	86.1
Determination-5	5937.4	54.2	85.8	6038.6	54.8	86.0	6046.0	54.4	87.0
Determination-6	5921.9	54.1	85.7	5956.7	54.6	85.1	6031.2	54.2	87.1
Assay mean (%)	86.1			85.6			86.7		
Assay STDEV	0.40			0.31			0.47		
Assay RSD (%)	0.47			0.36			0.54		
Pooled assay (%)	86.1								
Pooled STDEV	0.55								
Pooled RSD (%)	0.64								

### Robustness

The results of conscious small changes included a flow rate ± 0.1 mL/min and methanol (± 2.5%) were determined using RSD%. The RSD% was found to be < 2% in all cases, as shown in Tables 6 and 7. It is clear for man there is a reverse proportion between the retention time and the ratio of the organic modifier in the methanol. Where the retention time increases by decreasing the organic ratio, and vice versa. This assures the principle chromatographic rule “likes dissolve likes or likes attract likes”^11,13,34,35^.

**Table 6:** Change in the flow rate results (1.1–1.3 mL/min).

Standard replicate	Flow (1.2 mL)	Flow (1.3 mL)	Flow (1.1 mL)
Weight (mg)	54.2	54.2	54.2
1	5931.4	5510.0	6536.2
2	5944.4	5502.7	6515.0
3	5943.1	5498.4	6525.3
4	5925.0	5518.2	6544.8
5	5940.2	5512.4	6511.7
6	5915.2	5508.7	6582.4
Mean P.A	5933.2	5508.4	6535.9
Tests			
Weight (mg)	54.3	54.1	54.1
1	5931.1	5523.2	6511.8
2	5991.3	5538.2	6503.8
Mean P.A	5961.2	5530.7	6507.8
Assay (%)	86.0	86.3	85.5
Pooled assay (%)	85.9		
Pooled STDEV	0.36		
Pooled RSD (%)	0.42

**Table 7:** Change in methanol organic ratio results (54–66%).

Standard replicate	Methanol (60%)	Methanol (54%)	Methanol (66% mL)
Weight (mg)	54.2	54.2	54.2
1	5931.4	6781.2	5303.1
2	5944.4	6791.1	5299.4
3	5943.1	6786.0	5293.0
4	5925.0	6799.4	5287.8
5	5940.2	6785.7	5318.9
6	5915.2	6796.2	5290.2
Mean P.A	5933.2	6789.9	5298.7
Tests			
Weight (mg)	54.3	54.1	54.1
1	5931.1	6802.5	5312.9
2	5991.3	6807.7	5305.7
Mean P.A	5961.2	6805.1	5309.3
Assay (%)	86.0	86.1	86.1
Pooled assay (%)	86.1		
Pooled STDEV	0.06		
Pooled RSD (%)	0.06		

### Ruggedness

The results of conscious, major, and observable changes include day-to-day and column-to-column. The data was presented as shown in Tables 8 and 9. RSD% was found to be < 2% in all cases^11–13^.

**Table 8:** Day-to-day precision results.

Standard replicate	Day—1	Day—2	Day—3
Weight (mg)	54.2	54.5	54.4
1	5931.4	5991.5	5968.1
2	5944.4	5986.2	5950.8
3	5943.1	5994.6	5958
4	5925.0	5989.5	5954.7
5	5940.2	5988.9	5956.4
6	5915.2	5987.7	5960.9
Mean P.A	5933.2	5989.7	5958.2
Tests			
Weight (mg)	54.3	54.6	54.1
1	5931.1	6007.6	5928.2
2	5991.3	5992.6	5927.3
Mean P.A	5961.2	6000.1	5927.75
Assay (%)	86.0	85.7	85.8
Pooled assay (%)	85.8		
Pooled STDEV	0.14		
Pooled RSD (%)	0.16

**Table 9:** Column-to-Column precision results.

Standard replicate	Column—1	Column—2	Column—3
Weight (mg)	54.2	54.5	54.5
1	5931.4	6010.8	6006.5
2	5944.4	6008.6	6000.1
3	5943.1	6013.7	5991.4
4	5925.0	6030.6	5993.6
5	5940.2	6026.0	5996.1
6	5915.2	6014.8	6009.1
Mean P.A	5933.2	6017.4	5999.5
Tests			
Weight (mg)	54.3	54.6	54.6
1	5931.1	6010.7	6004.6
2	5991.3	5983.8	5999.7
Mean P.A	5961.2	5997.3	6002.2
Assay (%)	86.0	85.3	85.6
Pooled assay (%)	85.6		
Pooled STDEV	0.35		
Pooled RSD (%)	0.40		

### Stability of sample solution (at the HPLC rack)

The experimental results revealed that the tested solution of standard Tho could be given repeatable and precise peak response data over 48 h at room temperature, as in Table 10. Our investigation relegated the conclusion that the Tho is stable only at the special condition of − 20 °C as Anilanmert et al., conducted^22^ where they reported that Tho is not stable at room temperature or in a refrigerator. Also, Russo et al.^28^ showed very little solution stability at 27 °C, of Tho where they reported that the recovery percentage range from 5 to 75 μg/mL over 2 h was 42.2 ± 4.7% to 94.5 ± 2.7%.

**Table 10:** Stability of sample solution (at the HPLC rack) system suitability.

Time	P.A	Theoretical plates
Zero time	5931.4	5665
3 h	5915.8	5798
6 h	5919.3	5519
9 h	5952.0	5508
24 h	5939.8	5541
48 h	5927.2	5948
Pooled mean	5930.9	
Pooled STDEV	13.4	
Pooled RSD (%)	0.23	

### Specificity and selectivity

**Figure 4:**
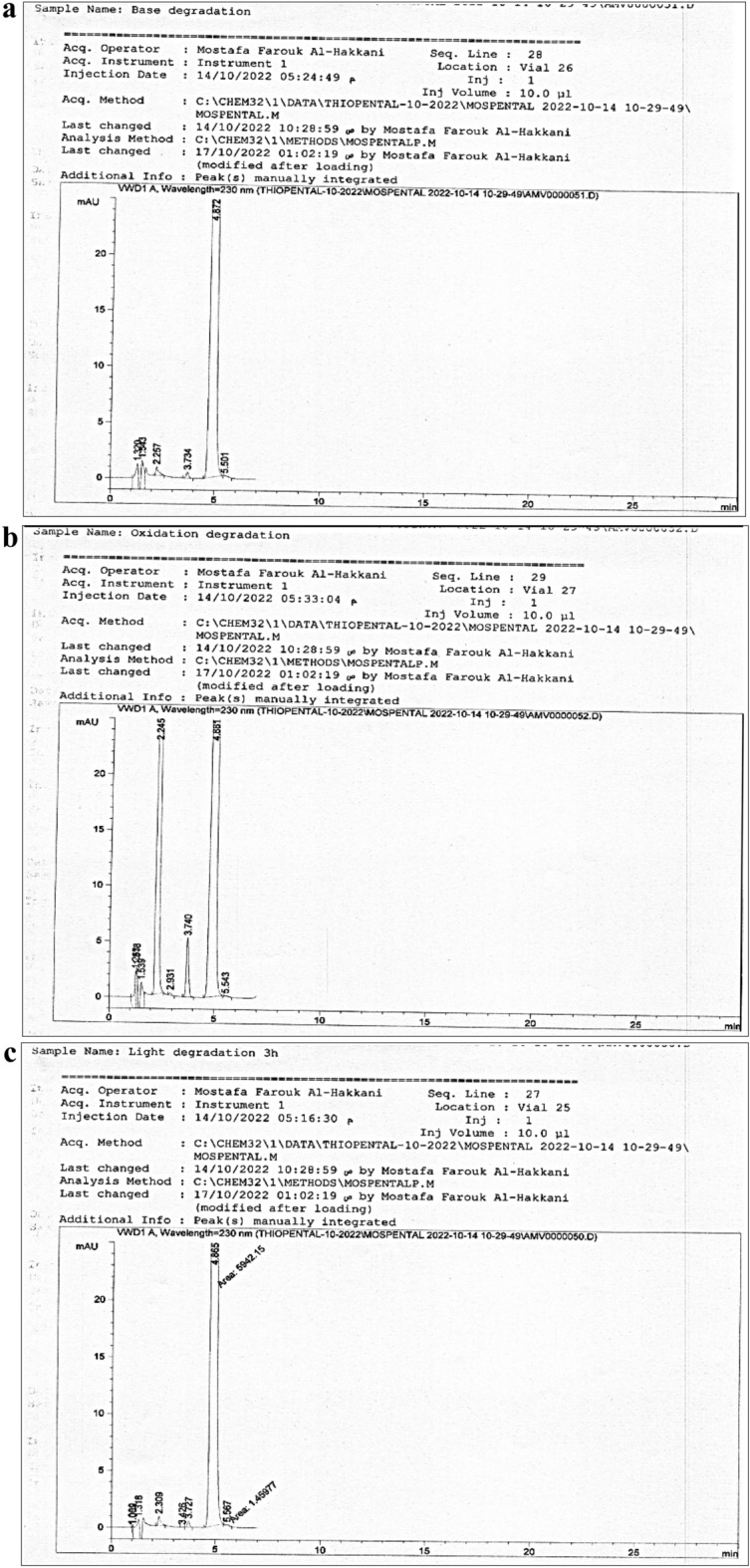
(**a**) Forced degradation using base hydrolysis 0.1 M NaOH for 30 min. (**b**) Forced degradation using H_2_O_2_ 3% w/v hydrolysis for 30 min. (**c**) Light forced degradation after 3 h.

The current method supplied us with highly specific data about the resolution and separation performance of the adjacent co-eluted peaks for the Tho principle peak with a resolution parameter of at least 2.77 as in Table 11 and Fig. 4a–c. It was observed that the acid hydrolysis on the Tho solution could not be applied, and a white precipitate was formed at the time of the addition without any signal response of the Tho after injection of the filtrate after centrifugation.

**Table 11:** Resolution factor at different forced degradation states.

Forced degradation item	Resolution
Acid degradation	It gives a white principate
Base degradation	2.92
Oxidation degradation	2.99
Light degradation	2.77

### Test of the validated method

#### Tho analysis of the different commercial dosage forms in the Egyptian local market

The Tho assay results of Thiopental UP Pharma 500 mg vials, and Thiopental Eipico 1.0 g vials revealed good results, manifested in the following Table 12. Where the method was found to be selective, specific, and resoluted for the Tho peak.

**Table 12:** Tho assay for the different local finished products.

Local name	Manufacturer	Strength	Assay (%)
Thiopental	UP pharma	500 mg	86.5
Thiopental	Eipico	1.0 g	84.2

#### Thiopental UP pharma 500 mg vials batch number (220741) after the constitution stability studies

The tabulated results in Table 13 confirmed the stability and validity of the use of the Tho solutions after constitution using WFI, saline 0.9% wt/v, and glucose 5% wt/v at 30 ± 2 °C and 5 ± 3 °C for 6 h and 24 h, respectively. Where the assay percentage was found to be within the acceptance criteria and did not exceed 3.0% from the starting assay at zero time. Also, the results manifested that the method did not affect the composition of the different initiators of the solvent on the retention time over the study.

**Table 13:** Tho assay after the constitution stability studies.

Item assay/solvent	WFI (%)	Saline 0.9% (%)	Glucose 5% (%)
Zero time	90.0	91.0	91.5
After 6 h at 30 ± 2 °C	90.3	88.5	90.9
After 24 h at 5 ± 3 °C	88.3	90.2	90.7

## Conclusion

The method was found to be accurate from concentration levels 350–600 µg/mL with high accuracy (100.4–101.4%), precise and repeatable for the same analyst over three days with intra–precision of 0.31%, inter–precision in the range 0.36–0.47% and inter-precision between two analysts of 0.16%. The linearity of the method was conducted in the range of 100–750 µg/mL with an excellent regression coefficient R^2^ = 0.9997. It proves its high capability to achieve the requirements of the chromatographic system suitability as follows: theoretical plates and column efficiency as 3412–5962, USP tailing at 0.96–1.07, and the RSD% of the peak areas at 0.05–0.74%. Finally, the selectivity and specificity of the current method were confirmed by realizing the minimum resolution between the thiopental principal peak and the most adjacent related impurity peak at 2.77. The validated method proved its performance capability in the separation of the thiopental principle peak from any other appearance-forced degradation peaks. The method revealed a wide accepted retention time range of 4.3–6.7 min, with an optimum retention time of 4.6 ± 0.3 min.

## Supplementary Information

Supplementary Table 1.

## Data Availability

All data generated or analyzed during this study are included in this article.
