# Supplementary material for: Rapidly, sensitive quantitative assessment of thiopental via forced stability indicating validated RP-HPLC method and its in-use stability activities
Source: Sci Rep. 2023 Jun 25;13:10294. doi: 10.1038/s41598-023-37329-0 (PMC10290985; doi:10.1038/s41598-023-37329-0)
Supplement: Supplementary file 1 — Supplementary Table 1. [file 41598_2023_37329_MOESM1_ESM.docx]

**Supplementary Table 1: Thiopental comparison of the different assay methods**

| **Type** | **Column** | **Reagent** | **Organic modifier** | **pH adjustment** | **Flow**  **mL/min** | **Detection**  **(min)** | **Accuracy (%)** | **LOD**  **µg/mL** | **Ref.** |
| --- | --- | --- | --- | --- | --- | --- | --- | --- | --- |
| **Voltammetry** | NA | Na_2_HPO_4_+ NaH_2_PO_4_ | NA | 10.5 | NA | 4-5 | 97.8-99.8 | 2.6 | [1] |
| **GC-FID** | 183 cm x 4 mm x 6 mm (glass) | NA | Isopropyl alcohol, n-hexane | NA | H_2_ 40  Air 425  N_2_ 45 | ≈3.0 | NA | NA | [2] |
| **GC-FID** | 180 cm x 2 mm (glass) | NA | Chloroform, ethyl acetate/acetic acid | NA | NA | < 2.0 | 97% | NA | [3] |
| **GC-FID** | 30 m × 0.32 mm x 0.25 μm | NA | Methanol | NA | He 2.0 | 1.97 | NA | NA | [4] |
| **GC–EI-MS** | Rtx-5MS, 30 m x 0.25 mm x 0.25 mm | NA | Methanol, acetonitrile, isopropanol, ethyl acetate | 5.5 | NA | 8.22 | 33% | 0.6 | [5] |
| **LC–MS/MS** | C18,100 ×  3.0 mm, 2.7  mm + Guard column, 4.0  mm × 2.0 mm | NaF | Acetone, acetonitrile, methanol, dichloromethane,  ethyl  acetate,  diethyl  ether,  n-hexane | 10.5-11 | 0.3 | 5.5 | 81.67±  4.22 | 3.47 | [6] |
| **HPCE** | Fused-silica capillary, 50 cm, 50 µm | Phosphate-borate  buffer + sodium  dodecyl sulphate | NA | 7.8 | NA | 4.0 | NA | NA | [7] |
| **HPTLC** | NA | Acetonitrile,  Methanol, chloroform | Hexane-dichloromethane-ethyl acetate | 5.0-6.5 | NA | NA | 87.46- 93.07 | 0.5 | [8] |
| **HPLC/UV** | Monolithic, Onyx C18,  100x4.6mm | Phosphate  buffer | Methanol and acetonitrile (40:5) | 2.7 | 4.0 | ≈10.6 | NA | 0.56 | [9] |
|  | Pre-column C18 + 150 mm x 4.6 mm, 5 µm | NA | NA | NA | NA | 12.04 | NA | NA | [4] |
|  | ODS C18, 150 x 4.6 mm, 4 µm | Trichloroacetic acid | Methanol, acetonitrile | 2.7 | 1.5 | 9.45 | 100.1-101.4 | < 0.2 | [10] |
|  | CLC-ODS, 150 x 6.0 mm, 5 µm | NA | Acetonitrile | NA | 1.0 | 5.8 | 97.4-101.4 | 0.05 | [11] |
|  | C18, 250 x 4.6 mm, 5 µm | Phosphate  buffer | Methanol | 6.5 | 1.0 | ≈5.0 | 104.64 | 0.02 | [12] |
|  | C18, 150 x 4.6 mm, 4 µm | Phosphate  buffer | Acetonitrile | 6.5 | 1.0 | ≈7.5 | 91.5-104.2 | 84 | [13] |
|  | CN, 150 x 3.9 mm, 5 µm + Post-column tube, 4 m × 0.25 mm | Iodine-azide | Acetonitrile | 7.8 | 1.0 | 3.6 | 92-116 | 0.005 | [14] |
|  | Pre-column, C18, 4 x 6 mm, 10 µm + 100 x 8 mm, 5 µm | NA | Acetonitrile | NA | 2.0 | 8.0 | 88 | 0.2 | [15] |
|  | Phenyl column, 150 x 4.6 mm, 5 µm | KH_2_PO_4_ | Acetonitrile | 4.0 | 1.0 | 4.9 | 98.8-101.3 | 0.317 | [16] |
|  | Pre-column, RP 8, 30 x 4 mm + RP 18 250 x 4 mm, 7 µm | NA | Methanol | 3.4 | 2.0 | 3.7 | NA | NA | [17] |
|  | NAIP column, 150 x 6 mm, 5 µm | Borate buffer | Acetonitrile | 7.0 | NA | ≈30 | 88.6-100 | 0.33 | [18] |
|  | C18- | NH_4_H_2_PO_4_ + Na_2_SO_4_ buffer | Methanol | 7.9 | 2.0 | ≈2.7 | NA | NA | [19] |
|  | RP 8, 250 x 4.6 mm, 10 µm | NA | Methanol | NA | 2.0 | ≈4.0 | 93.3-100.7 | 0.018 | [20] |
|  | C8, 250 x 8 mm, 5 µm | Phosphate buffer | Acetonitrile | 6.4 | 1.2 | 10.0 | NA | NA | [7] |
|  | C8, 110 x 4.6 mm | Phosphate buffer | Acetonitrile | 6.2 | 1.0 | ≈4.3 | NA | NA | [21] |
|  | RP C18, 150 x 3.9 mm | (NH_4_)_2_HPO_4_ | Methanol | 8.0 | 1.0 | 9.1 | 78-87 | 0.03 | [22] |
|  | C18, 300 x 3.9 mm | KCI | Methanol | 2.0 | 2.0 | 3.8 | 101 | 0.03-0.09 | [23] |
|  | C18, 125 x 4.0 mm, 5 µm + guard column, 4.0 x 4.0 mm, 5 µm | Triethylammonium  phosphate buffer | Acetonitrile | 3.0 | 2.0 | 3.46 | NA | 0.23 | [24] |
|  | Pre-column , RP 18, 30 X 4 mm + RP 18 column, 25 x 4.0 mm, 7 µm | NaH_2_P0_4_ buffer | Acetonitrile | 4.6 | 1.0 | 6.8 | 80-92 | NA | [25] |
|  | Pre-cohnnn, C18, 50 x 4.6 mm, 30-40 µm + C6 column, 200 x 4.6 mm, 5 µm | Sodium acetate | Acetonitrile | 3.6 | 1.5 | 9.7 | 98-101.8 | NA | [26] |
|  | RP C18, 100 X 3 mm, 5 µm | NA | Acetonitrile | NA | 0.3 | ≈14 | 94 | 0.01 | [27] |
|  | RP C18, 300 X 4 mm, 10 µm | Perchlorate buffer | Acetonitrile | 2.9 | 1.5 | 8.3 | 93.6-105.7 | 100 | [28] |
|  | Guard pre-column C18, 10 µm + C8 column, 100 X 8 mm, 10 µm | Phosphate  buffer-tetrahydrofuran | Methanol | 7.73 | 2.5 | 8.54 | 84.4-88.6 | 1.25 | [29] |
|  | Pre-column C18, 50 x 4 mm + C18 column, 300 X 4 mm, 5 µm | Potassium phosphate | Methanol | 4.4 | 1.7 | 8.9 | 97-103 | 10 | [30] |
|  | ODS C18, 300 X 3.9 mm, 10 µm | Phosphate buffer -  tetrahydrofuran | Acetonitrile | 7.8 | 2.0 | 12.0 | 96-104 | 0.1-0.2 | [31] |

* NA: not applicable.

1. Ali AM, Farghaly OA, Ghandour M: **Determination of thiopentone sodium in aqueous and biological media by cathodic stripping voltammetry**. *Anal Chim Acta* 2000, **412**(1-2):99-110.

2. Van Hamme M, Ghoneim M: **A sensitive gas chromatograph assay for thiopentone in plasma**. *Br J Anaesth* 1978, **50**(2):143-145.

3. Külpmann W: **Gas-chromatographic method for the determination of thiopental in serum**. *Z Anal Chem* 1982, **311**(4):409-409.

4. Colatutto A, Zaglia R, Isola L, Bearzatto S, Marcon B, Sala P: **Comparison between a high-perfomance liquid chromatographic method and a gas chromatographic method for the quantification of serum thiopental for the diagnosis of brain death in critically ill patients**. *Riv Ital Med Lab J* 2012, **8**(3):162-167.

5. Adamowicz P, Kała M: **Simultaneous screening for and determination of 128 date-rape drugs in urine by gas chromatography–electron ionization-mass spectrometry**. *Forensic Sci Int* 2010, **198**(1):39-45.

6. Anilanmert B, Çavuş F, Narin I, Cengiz S, Sertler Ş, Özdemir AA, Açikkol M: **Simultaneous analysis method for GHB, ketamine, norketamine, phenobarbital, thiopental, zolpidem, zopiclone and phenytoin in urine, using C18 poroshell column**. *J Chromatogr B* 2016, **1022**:230-241.

7. Meier P, Thormann W: **Determination of thiopental in human serum and plasma by high-performance capillary electrophoresis—micellar electrokinetic chromatography**. *J Chromatogr A* 1991, **559**(1-2):505-513.

8. Sanganalmath PU, Nagaraju PM, Mohan BM: **HPTLC method for the assay of thiopental in post-mortem blood in a fatal case of suicide**. *J Pharm Biomed Anal* 2013, **80**:89-93.

9. Beril A, Ibrahim N, Ibrahim A, Munevver A: **Fast chromatographic screening method for 7 drugs of potential threat in drug facilitated crimes**. *Pak J Pharm Sci* 2018, **31**:231-235.

10. Coppa G, Testa R, Gambini AM, Testa I, Tocchini M, Bonfigli AR: **Fast, simple and cost-effective determination of thiopental in human plasma by a new HPLC technique**. *Clin Chim Acta* 2001, **305**(1):41-45.

11. Hosotsubo H: **Determination of thiopental in human serum and plasma by reversed-phase high-performance liquid chromatography**. *Chromatographia* 1988, **25**(10):887-890.

12. Elmansi H, Elsayed N, Belal F: **Novel approach for chromatographic separation of atracurium and thiopental using factorial model**. *Microchem J* 2021, **170**:106751.

13. Najafi M, Pashabadi A: **Ultrasound-assisted surfactant-enhanced emulsification microextraction-HPLC determination of low-levels of thiopental in serum and urine samples**. *J Rep Pharm Sci* 2015, **4**(1):30.

14. Zakrzewski R, Ciesielski W: **Determination of thiopental in urine sample with high-performance liquid chromatography using iodine–azide reaction as a postcolumn detection system**. *J Chromatogr B* 2005, **824**(1):327-332.

15. Russo H, Allaz JL, Bressolle F: **High-performance liquid chromatographic assay for thiopental in human plasma: Application to pharmacokinetic studies**. *J Chromatogr B: Biomed Sci Appl* 1997, **694**(1):239-245.

16. King D, Stewart J, Venkateshwaran T: **HPLC determination of propofol-thiopental sodium and propofol-ondansetron mixtures**. *J Liq Chromatogr Relat Technol* 1996, **19**(14):2285-2294.

17. Sauerbrey R, Reiber H: **Analysis of thiopental in serum and cerebrospinal fluid by HPLC**. *Fresenius' J Anal Chem* 1982, **311**(4):412-412.

18. Kuroda N, Inoue K, Mayahara K, Nakashima K, Akiyama S: **Application of 3-(1, 8-Naphthalimido) Propyl-Modified Silyl Silica Gel as a Stationary Phase In High Performanc Liquid Chromatography of Barbiturates and Diastereomeric Compounds**. *J Liq Chromatogr Relat Technol* 1996, **19**(17-18):2867-2881.

19. Sharman JR, Ahern KM: **A simple method for determining thiopental in plasma**. *J Anal Toxicol* 1983, **7**(1):37-39.

20. Gruhl H, Mayer H: **Simple and rapid determination of thiopental in serum by HPLC**. *J Clin Chem Clin Biochem* 1984, **22**(5):385-388.

21. Schmid RW, Wolf C: **Simultaneous determination of thiopental and its metabolite, pentobarbital, in blood by high-performance liquid chromatography and post-column photochemical reaction**. *J Pharm Biomed Anal* 1989, **7**(12):1749-1755.

22. Mangin P, Lugnier A, Chaumont A: **A polyvalent method using HPLC for screening and quantification of 12 common barbiturates in various biological materials**. *J Anal Toxicol* 1987, **11**(1):27-30.

23. Christensen JH, Andreasen F: **Determination of thiopental by high pressure liquid chromatography**. *Acta Pharmacol Toxicol* 1979, **44**(4):260-263.

24. Hannak D, Scharbert F, Kattermann R: **Stepwise binary gradient high-performance liquid chromatographic system for routine drug monitoring**. *J Chromatogr A* 1996, **728**(1-2):307-310.

25. Björkman S, Idvall J: **A high-performance liquid chromatographic method for methohexital and thiopental in plasma or whole blood**. *J Chromatogr B: Biomed Sci Appl* 1984, **307**:481-487.

26. Premel-Cabic A, Turcant A, Cailleux A, Allain P: **Micromethod for determination of thiopental in human plasma by high-performance liquid chromatography**. *J Chromatogr B: Biomed Sci Appl* 1983, **276**:451-455.

27. Celardo A, Bonati M: **Determination of thiopental measured in human blood by reversed-phase high-performance liquid chromatography**. *J Chromatogr B: Biomed Sci Appl* 1990, **527**:220-225.

28. Marty P, Arnoux P, Cano J: **Micromethod for determination of thiopental in biological fluids by high performance liquid chromatography. Application to therapeutic follow-up and pharmacokinetic study**. *Anal Lett* 1988, **21**(12):2241-2252.

29. Avram MJ, Krejcie TC: **Determination of sodium pentobarbital and either sodium methohexital or sodium thiopental in plasma by high-performance liquid chromatography with ultraviolet detection**. *J Chromatogr B: Biomed Sci Appl* 1987, **414**:484-491.

30. Houdret N, Lhermitte M, Lalau G, Izydorczak J, Roussel P: **Determination of thiopental and pentobarbital in plasma using high-performance liquid chromatography**. *J Chromatogr B: Biomed Sci Appl* 1985, **343**:437-442.

31. Shiu GK, Nemoto EM: **Simple, rapid and sensitive reversed-phase high-performance liquid chromatographic method for thiopental and pentobarbital determination in plasma and brain tissue**. *J Chromatogr B: Biomed Sci Appl* 1982, **227**(1):207-212.
